# Proton-Translocating Nicotinamide Nucleotide Transhydrogenase: A Structural Perspective

**DOI:** 10.3389/fphys.2017.01089

**Published:** 2017-12-19

**Authors:** Qinghai Zhang, Pius S. Padayatti, Josephine H. Leung

**Affiliations:** Department of Integrative Structural and Computational Biology, The Scripps Research Institute, La Jolla, CA, United States

**Keywords:** hydride transfer, lipidic cubic phase, membrane protein, nucleotide binding, NADPH, proton channel, transhydrogenase, X-ray crystallography

## Abstract

Nicotinamide nucleotide transhydrogenase (TH) is an enzyme complex in animal mitochondria and bacteria that utilizes the electrochemical proton gradient across membranes to drive the production of NADPH. The enzyme plays an important role in maintaining the redox balance of cells with implications in aging and a number of human diseases. TH exists as a homodimer with each protomer containing a proton-translocating transmembrane domain and two soluble nucleotide binding domains that mediate hydride transfer between NAD(H) and NADP(H). The three-domain architecture of TH is conserved across species but polypeptide composition differs substantially. The complex domain coupling mechanism of TH is not fully understood despite extensive biochemical and structural characterizations. Herein the progress is reviewed, focusing mainly on structural findings from 3D crystallization of isolated soluble domains and more recently of the transmembrane domain and the holo-enzyme from *Thermus thermophilus*. A structural perspective and impeding challenges in further elucidating the mechanism of TH are discussed.

## Introduction

Nicotinamide nucleotide transhydrogenase (TH) is a key enzyme residing in mitochondrial inner membrane and bacterial cytoplasmic membrane that helps to maintain cellular redox balance through the following reaction:

$$\begin{array}{l} H^{{+}_{out}}+NADP^{+}+NADH\;↔H^{{+}_{in}}+NADPH+NAD^{+} \end{array}$$

In this reaction, proton motive force across the membrane is utilized to drive hydride transfer from NADH to NADP^+^, resulting in the generation of NADPH under most physiological conditions (Jackson, 2003). TH is estimated to account for the generation of about 40% of NADPH in *E. coli* and an even higher percentage in the mitochondria of vertebrates (Sauer et al., 2004; Rydström, 2006a). Mitochondria utilize NADPH to maintain high levels of glutathione, a key component in cellular defense against the accumulation of reactive oxygen species which are implicated in many pathological conditions, such as cancer, diabetes, hypertension, heart disease, Alzheimer's, and Parkinson's diseases, as well as cell death and aging (Rydström, 2006b; Albracht et al., 2011; Gameiro et al., 2013; Ghosh et al., 2014; Lopert and Patel, 2014; Picard et al., 2015; Ho et al., 2017). Animal model and clinical investigations have indicated that some of these conditions can be attributed to the dysfunction or misexpression of TH (Heiker et al., 2013; Nickel et al., 2015; Roucher-Boulez et al., 2016; Leskov et al., 2017; Santos et al., 2017; Scott et al., 2017). Mutations in the enzyme have also been shown in patients with glucocorticoid deficiency syndrome (Meimaridou et al., 2012; Fujisawa et al., 2015; Weinberg-Shukron et al., 2015).

**Figure 1:**
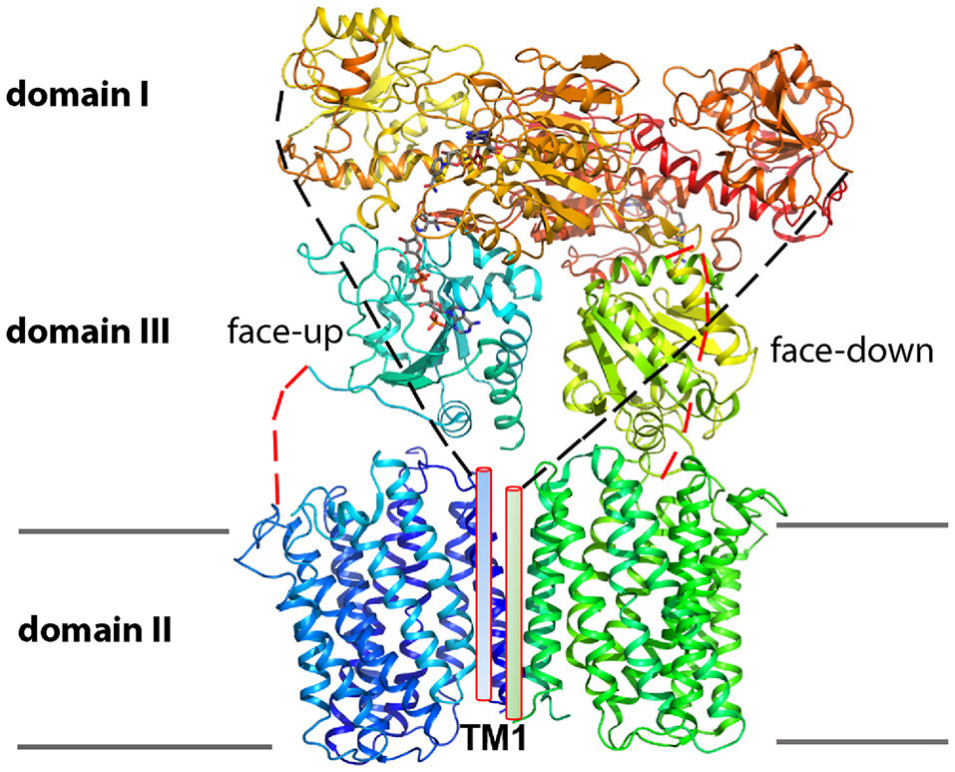
Architecture of holo-TH. TH varies in polypeptide compositions in different species but displays an overall conserved domain architecture. The cartoon is based on a holo-TH crystal structure from *T. thermophilus* (PDB: 4O9U), shown as a dimer with three domains. Domain I and domain III protrude from membrane-intercalated domain II (membrane boundaries in gray line), with domain III placed in the middle. A linker of 14–19 amino acids (red dashed lines), which is not revealed in 4O9U, connects domain II and III in all TH homologs. Domain I is expressed as a single polypeptide in *T. thermophilus*, but in mitochondrial TH it is fused with domain II through a linker (black dashed lines) and an additional TM1 (rod).

TH is a complex multi-domain protein arranged as a dimer, where each protomer has two soluble domains and a transmembrane domain (Figure 1) (Leung et al., 2015). Extensive biochemical and structural characterizations have essentially established the domain architecture, the catalytic reaction sites as well as the proton translocating pathway as will be discussed in this review. The soluble domains are located in the mitochondrial matrix or prokaryotic cytosol and include a 40 kDa NAD(H)-binding domain (domain I) and a 20 kDa NADP(H)-binding domain (domain III) which together catalyze the hydride transfer reaction. The 40 kDa transmembrane domain (domain II) forms the proton translocation channel. This three-domain architecture is universally conserved, suggesting a common mechanism for function. However, TH topologies and sequences vary substantially among species. For example, human TH is encoded by a single polypeptide chain, whereas TH from *E. coli* and *T. thermophilus* are encoded by two and three polypeptide chains, respectively. In *E. coli* TH, domain I and the first four transmembrane helices (TM 1–4) of domain II are encoded by one gene α, whereas in *T. thermophilus, two* separate genes (α1 and α2) encode domain I and the first three TM helices (TM 2–4) of domain II; the remaining TM helices (TM 6–14) of domain II together with domain III in both species are encoded by gene β. Of note, domain II differs in the number of TM helices in different species, for example, with 14 for human, 13 for *E. coli* and 12 for *T. thermophilus*. To date, most functional and structural characterizations have been performed on TH from lower organisms (Pedersen et al., 2008; Jackson, 2012), and it will be interesting to learn how function is conducted in TH from higher organisms with evolved sequences, and how mutations in human TH cause disease.

Mechanistic understanding of TH has been advanced significantly by the structure determinations of isolated soluble domains and more recently of the transmembrane domain and the holo-enzyme from *T. thermophilus* (Leung et al., 2015; Padayatti et al., 2017). The architecture of the three domains of TH is explicitly established with the NADP(H)-binding domain III sandwiched between domains I and II and showing significant conformational mobility. A novel mechanism of domain III swiveling has been proposed for its communication with each of the other two domains alternately within the biological dimer (Jackson et al., 2015). This manuscript will review progress in determining TH structure, new mechanistic insights that have resulted, and some questions that remain in order to fully elucidate the conformational dynamics and mechanism by which proton translocation is coupled to hydride transfer events that occur remotely.

## Structures of nucleotide binding domains I and III

Individual crystal structures of the two soluble domains of TH showed overall similarity across multiple species including human, bovine, *E. coli*, and *R. rubrum* (Prasad et al., 1999, 2002; White et al., 2000; Sundaresan et al., 2003; Johansson et al., 2005). Domain I is present as a dimer in solution and always crystallized as such, with the dimeric interface stabilized by a swapping beta-hairpin structure and two helices. Each monomer of domain I has two subdomains with the characteristic nucleotide binding Rossmann fold (Rossmann et al., 1974); yet only one of the subdomains actually binds NAD(H). Thus, in the domain I dimer that adopts a two-fold axis symmetry, the two NAD(H) binding sites are situated on opposite sides. In comparison, domain III is about half the size of domain I, also adopts the Rossmann fold for NADP(H) binding, but only exists as a monomer in solution. Of note, the orientation of NADP(H) in the binding pocket of domain III is flipped relative to that of NAD(H) in domain I.

Local conformational changes are observed in nucleotide-binding regions in domain I and domain III. Various modes of NAD(H) binding such as open, intermediate, and closed forms have been demonstrated, both in domain I structures and the apo structures (Prasad et al., 2002; Johansson et al., 2005). In *E. coli* TH holoenzyme, the dissociation constant (Kd) for NAD^+^ is 100–500 μM and that for NADH is 50 μM, comparable to values for the isolated domain I (Bizouarn et al., 2005). Domain III has hitherto only been crystallized in the presence of NADP(H), with loop D either in open or closed conformation (Prasad et al., 1999; White et al., 2000; Sundaresan et al., 2003). Notably, NADP(H) has extremely high binding affinity with the isolated domain III (apparent Kd value < nM) (Diggle et al., 1995; Fjellstrom et al., 1997; Peake et al., 1999), which is partly attributed to adjacent loop E folding over NADP(H) like a lid. Interestingly, for domain III in the intact *E. coli* TH, the binding affinity of NADP(H) is orders of magnitude weaker: the Kd values are 16 μM for NADP^+^ and 0.87 μM for NADPH (Bizouarn et al., 2005). It is not understood why the binding affinity of NADP(H) differs so widely between isolated domain III and the intact TH, but existing data suggest that the NADP(H)-binding site is affected by protein-protein interactions between domain III and other domains. For completion of the TH catalytic cycle, all nucleotide reactants must bind and products must dissociate from the binding sites. The holoenzyme likely uses protein conformational changes to alter nucleotide binding affinities and move the reaction (Jackson, 2012).

## Structures of domain I and domain III in complex

**Figure 2:**
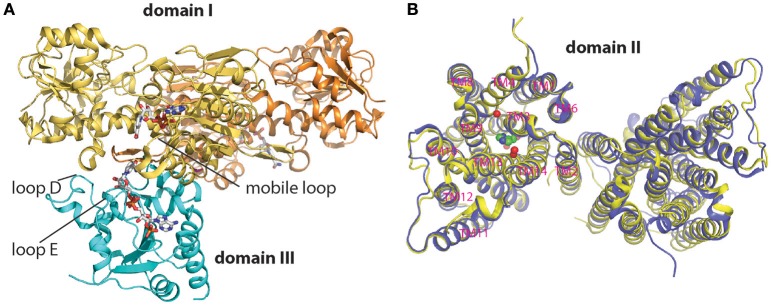
Reaction catalysis and proton-translocating sites in TH. **(A)** Hydride transfer between the proximate NAD(H) and NADP(H) binding sites of domains I and III. Shown is a nucleotide-bound (sticks), trimeric (domain I)_2_:(domain III) structure of TH from *T. thermophilus* (PDB: 4J16), with the NADP(H) binding site in domain III (cyan) facing up. Flexible loops involved in ligand binding are indicated. **(B)** Proton translocation in domain II. Domain II dimerization (from *T. thermophilus*) is mediated by TM2-TM2 interaction. Displacement of the second subunit is observed by the alignment of the two structures (PDB: 5UNI, blue; 4O93, yellow) solved under different pH conditions. Crystallographically solved water molecules (red balls) and the channel gating histidine residue (His42^α2^ on TM3, green sphere) are shown inside the proton channel within one subunit of 5UNI. Structures shown represent a view from the cytoplasmic side.

Co-crystallization of isolated domains I and III either from *R. rubrum* or *T. thermophilus* consistently yields a heterotrimeric complex at a 2:1 ratio (Figure 2A) (Cotton et al., 2001; Mather et al., 2004; Sundaresan et al., 2005; Bhakta et al., 2007; Leung et al., 2015). This stoichiometry agrees with findings in solution (Venning et al., 1998, 2001). The Kd of domain III binding to the domain I dimer is < 60 nM (Venning et al., 2001), while a second domain III binds with significantly lower affinity (Kd about 10^−5^ M) (Quirk et al., 1999). In the heterotrimeric structures, the two domain I components adopt the same two-fold axis as in the isolated domain I dimer structure, with similar protein fold and contact regions between the polypeptides; in addition a domain III is placed underneath one domain I subunit with the NADP(H) binding site face up. However, using the same two-fold axis to model a second “face-up” domain III into the void space under the other domain I subunit results in a steric clash between the two domain III subunits. This leaves a question of how the second domain III is made to fit in the dimeric structure of the holo-TH.

The heterotrimeric structures of domain I and domain III reveal proximity between the NAD(H) and NADP(H) binding sites (Figure 2A). In these structures, the single copy of domain III is inserted into the NAD(H)-binding cleft of one of the domain I subunits, and the flipped orientation of NADP(H) in domain III apparently facilitates a direct hydride transfer between the nicotinamide rings of NAD(H) and NADP(H) when the two ligands get close. Local conformational changes in the NAD(H) binding site as well as relative movement between the two subdomains within domain I appear to cause a “distal-to-proximal” motion of bound NAD(H) (Mather et al., 2004).

## Structures of transmembrane domain II

Structural determination of the transmembrane domain of TH has recently been achieved by the use of lipidic cubic phase (LCP) crystallization (Leung et al., 2015; Padayatti et al., 2017). The LCP is a viscous bilayer matrix that is thought to better stabilize membrane proteins than detergents and more frequently results in a tighter and layered crystal packing (Caffrey and Cherezov, 2009). LCP crystallization succeeded for a truncated *T. thermophilus* TH domain II construct (containing two hydrophobic polypeptide chains: subunit α_2_ and a truncated version of subunit β from which the soluble domain III was removed from the C-terminus), using 1-(8*Z*-pentadecenoyl)-*rac*-glycerol (MAG 8.7) as the host lipid. Compared to the commonly used monoolein (MAG 9.9), MAG 8.7 has a shorter alkyl chain and tends to form a more fluidic cubic phase.

Domain II was crystallized at pH 8.5 and 6.5, respectively, and the solved crystal structures both appear as a dimer with TM2-TM2 association at the interface (Figure 2B). The two structures are quite similar overall, although alignment shows a slight displacement of the second domain II monomer, suggesting a degree of conformational flexibility to the association; yet at this stage no evidence is found for a substantial conformational change related to channel opening or closure. The crystal structures also reveal a putative channel surrounded by six TM helices (TM 3, 4, 9, 10, 13, and 14). It should be noted that these channel-forming helices and the TM2 at the dimeric interface are highly conserved among species, suggesting a common proton translocation pathway and possibly a similar mode of dimerization despite considerable homolog variation in the organization of this domain II (e.g., the presence of extra TM1 and TM5 in human TH).

The pH 6.5 crystal structure solved at a resolution of 2.1 Å revealed water molecules inside the TM helix bundles, which, in combination with molecular dynamics (MD) simulations, allows a clearer interpretation of the proton translocation channel and its key functional residues (Figure 2B) (Padayatti et al., 2017). A cluster of polar residues including a central histidine (His42^α2^) form an extended H-bonded network together with two bound water molecules in the cytoplasmic half of the channel, and this connection likely provides a proton conduit to the cytoplasmic side. A central role for His42^α2^ in proton translocation by TH is consistent with extensive biochemical characterizations; the central histidine is conserved in other TH species although its position may shift (Olausson et al., 1995; Rydström et al., 1998; Bragg and Hou, 2001; Yamaguchi et al., 2002). MD simulation showed that a water conduit is formed only when His42^α2^ is protonated. Gating of the isolated channel appears to be controlled by His42^α2^ protonation and also a distinct hydrophobic barrier region spanning about 7.5 Å below His42^α2^ in the middle of the channel. The presence of this long stretch of hydrophobic region for proton gating is remarkable for TH, in contrast to the water penetration through thin hydrophobic layers known for some other proton channels (Pielak and Chou, 2011; Takeshita et al., 2014; Thomaston et al., 2017). During MD simulations, a transient water wire is formed connecting His42^α2^ and Glu221^β^ through the hydrophobic barrier, and the presence of the water wire coincides with a conformational change of Thr214^β^ side chain located slightly below His42^α2^. Mutation of Thr214^β^ to Ala causes >90% loss of channel-coupled enzymatic activity, biochemically supporting the proposed role of Thr214^β^ in proton translocation (Padayatti et al., 2017).

The domain II structure further shows formation of a salt bridge between Asp202^β^ on the cytosolic end of TM13 and Arg254^β^ at the start of the linker region connecting domain II and domain III. This linkage is positioned on the membrane surface adjacent to the entrance of the proton channel. The Asp-Arg salt bridge at equivalent positions in *E. coli* TH (Asp213^β^ and Arg265^β^) has been postulated and implicated as important for both proton translocation in domain II and NADP(H) binding in domain III (Althage et al., 2001; Bragg and Hou, 2001; Yamaguchi et al., 2002). Since in TH of various species the sequence of domain II always exists in conjunction with domain III (Figure 1), regulation of domain III movement may be directly linked to domain II and the proton channel activity, and vice versa.

## Holo-TH structures

A holo-TH structure from *T. thermophilus* has recently been solved in detergent micelles, albeit only at 6.93 Å resolution (Figure 1) (Leung et al., 2015). This low resolution structure seemingly reflects substantial conformational flexibility of the complex protein and the significant challenge in identifying suitable crystallization conditions. The soluble domain I dimer and membrane-intercalated domain II components in the holo-TH are roughly aligned with individually obtained domain structures. A remarkable realization from this structure is the asymmetric orientation of two domain III subunits, one being flipped ~180°C relative to the other. This distinct domain III arrangement in the dimeric TH brings one NADP(H) binding site close to the NAD(H) binding site of domain I, as shown in the heterotrimeric (domain I)_2_-domain III structures, while the other NADP(H) binding site faces down to approach the channel surface of domain II. The two orientations of domain III are supported by disulfide cross-linking of inserted cysteine pairs on separate domains (Leung et al., 2015). The flipped orientation of domain III also appears to coincide with that observed in the crystal packing of (domain I)_2_-domain III structures where a crystallographic symmetry mate of domain III is similarly facing down (Sundaresan et al., 2005; Bhakta et al., 2007). Consistent with crystal structures, cryogenic electron microscopy (EM) images (~18 Å) of the holo-TH confirm an asymmetric organization of TH dimers in non-crystalline conditions (Leung et al., 2015). Cryo-EM structures also suggest that one subunit of domain III is more disordered than the other as evidenced by much weaker electron density. While a further high-resolution structure of holo-TH is warranted, based on current structural findings, the two copies of domain III have been proposed to play distinct functional roles in coupling to proton translocation in domain II and in hydride transfer from/to domain I (Jackson et al., 2015).

## Perspective and concluding remarks

The mechanistic understanding of mitochondria TH has benefited from extensive structural and biochemical characterizations of isolated domains and homolog proteins, particularly those from lower organisms. The use of stably expressed and purified TH from *T. thermophilus* has resulted in the recent structural determinations of the membrane-intercalated domain II and holo-TH. For the latter, higher-resolution structural determinations of the large multidomain membrane protein complex remain a significant challenge due to its significant conformational flexibility/heterogeneity. The solved holo-TH structure in detergents is of low resolution and lacks information for the linker (14–19 amino acids present in all TH) that connects domain II and domain III, and which is supposedly important to mediate the two domain interactions and the rotation of domain III as well. Modeling of human TH structure based on homologs is challenging given the significant sequence disparity, especially for domain II because of the presence of extra TM segments in the human enzyme (Metherell et al., 2016). The fusion of all three domains in a single polypeptide chain in human TH may impact its conformational landscape and energetics as well, although a common mechanism of the TH-catalyzed enzymatic reactions is expected for different species. At present, our understanding of the dynamic domain coupling and movements in TH is still very limited and mostly hypothetical. Structural insights into the mechanism of a dynamic TH will thus require the structural determinations of the protein complex in many conformational states. Recent technological breakthroughs in X-ray crystallography and cryo-EM have helped solve structures of many difficult-to-study systems. The use of novel lipid bilayer mimetics such as LCP (Caffrey, 2015) and nanodiscs (Denisov and Sligar, 2017) in the structural determinations of membrane proteins has gained popularity in addition to the traditional use of detergents. With the application and integration of these advanced techniques with new tools in TH studies, we anticipate that a comprehensive and detailed structural and mechanistic understanding can be achieved for this fundamentally important enzyme in many kingdoms of life.

## Author contributions

All authors listed have made a substantial, direct and intellectual contribution to the work, and approved it for publication.

### Conflict of interest statement

The authors declare that the research was conducted in the absence of any commercial or financial relationships that could be construed as a potential conflict of interest.
